# Longitudinal Assessment of FT3 to FT4 Conversion Ratio in Predicting the Efficacy of First-Line Pembrolizumab-Based Therapy in Advanced Non-Small Cell Lung Cancer: A Propensity-Score Matching Analysis of Data from the National Drug Monitoring Agency

**DOI:** 10.3390/curroncol31120564

**Published:** 2024-12-01

**Authors:** Fabrizio Nelli, Enzo Maria Ruggeri, Marta Schirripa, Antonella Virtuoso, Diana Giannarelli, Armando Raso, Daniele Remotti, Agnese Fabbri

**Affiliations:** 1 Department of Oncology and Hematology, Medical Oncology Unit, Central Hospital of Belcolle, 01100 Viterbo, Italy; 2 Biostatistics Unit, Scientific Directorate, Fondazione Policlinico Universitario A. Gemelli, IRCCS, 00168 Rome, Italy; 3 Department of Oncology and Hematology, Thoracic and Interventional Radiology, Central Hospital of Belcolle, 01100 Viterbo, Italy; 4 Department of Oncology and Hematology, Pathology Unit, Central Hospital of Belcolle, 01100 Viterbo, Italy

**Keywords:** non-small-cell lung cancer, fT3/fT4, immune checkpoint blockade, pembrolizumab, chemotherapy, first-line therapy, efficacy

## Abstract

Baseline thyroid function, as measured by the fT3 to fT4 ratio, has been shown to influence the prognosis of advanced cancer patients receiving active treatments. Although immune checkpoint blockade can alter the balance of thyroid hormones, this interaction has not been thoroughly investigated. The present research sought to determine whether changes in the fT3/fT4 ratio could affect the survival outcomes of patients with advanced non-small cell lung cancer (NSCLC) who were undergoing pembrolizumab-based therapies. This study included patients with metastatic NSCLC who received pembrolizumab as upfront treatment, either alone or in combination with platinum-based chemotherapy. Relevant data were gathered before the start (time point 1) and after 12 weeks (time point 2) of treatment. From April 2018 to May 2023, we enrolled 258 eligible patients, 156 (60.5%) and 102 (39.5%) of whom were treated with single-agent or combination therapy, respectively. We stratified patients into two groups based on baseline fT3 and fT4 values [euthyroid cohort defined by fT3 and fT4 both within the normal range vs. euthyroid sick syndrome cohort defined by low fT3 and/or fT4 levels]. We examined the differences in progression-free survival (PFS) and overall survival (OS) by univariate and multivariate analyses. After applying propensity-score matching, we considered 88 relevant cases in each cohort. Longitudinal comparison of fT3/fT4 ratios showed a significant increase in the median value after pembrolizumab-based therapy (*p* < 0.001). We computed ROC curves to analyze the correlation between fT3/fT4 ratios and survival outcomes. The relative AUC values were not viable in predicting a positive outcome at the first time point. Conversely, assessment at the second time point revealed a significant association with PFS [AUC 0.82 (95% CI 0.75–0.89), *p* < 0.001] and OS [AUC 0.81 (95% CI 0.75–0.88), *p* < 0.001]. After a median follow-up of 20.2 (95% CI 16.2–24.2) months, the median PFS for the low and high fT3/fT4 ratio groups was 4.1 (95% CI 3.0–5.1) and 15.3 (95% CI 10.3–20.1) months, respectively (*p* < 0.001). The median OS for the low and high fT3/fT4 ratio groups was 6.7 (95% CI 4.9–8.5) and 19.6 (95% CI 16.4–22.8) months, respectively (*p* < 0.001). The multivariate analysis revealed that a low fT3/fT4 ratio was independently associated with shorter PFS [HR 2.51 (1.66–3.78); *p* < 0.001] and OS [HR 2.18 (1.43–3.34); *p* < 0.001]. After the optimal weighting of prognostic factors according to thyroid function impairment, the fT3/fT4 ratio at baseline did not affect the survival of patients receiving immune checkpoint blockade for advanced NSCLC. Patients with an increased fT3/fT4 ratio experienced a significantly decreased risk of disease progression and mortality. The longitudinal assessment of fT3/fT4 ratio may play a predictive role in this specific therapeutic setting.

## 1. Introduction

The advances in cancer immunology over the past decade have significantly changed the treatment approach for solid tumors [1]. Immune checkpoint blockade has become a standard option, replacing or complementing cytotoxic chemotherapy and targeted therapies [2]. Immune checkpoint inhibitors (ICIs) have been widely used to treat advanced non-small cell lung cancer (NSCLC), and they are now recommended as upfront treatment for all patients with this condition [3]. However, while an improvement has been observed, the long-term survival rate with immune checkpoint blockade in late-stage NSCLC remains below 30% [4,5,6]. Additionally, the potential for immune-related side effects and the high cost of these treatments make the overall risk–benefit ratio unsatisfactory [7]. There is a growing recognition that identifying predictive biomarkers could improve the therapeutic index of immune checkpoint blockade by distinguish between responders and non-responders [8]. The only biomarkers approved by regulatory authorities are tumor-based features, such as programmed death ligand 1 (PD-L1) expression on tumor cells, tumor mutational burden (TMB), and DNA repair defects including deficient mismatch repair (dMMR) and high microsatellite instability (MSI-H) [9]. In parallel with efforts to identify tumor-related biomarkers, clinical research has focused on developing blood-based biomarkers. Soluble biomarkers could allow for easy, repeatable, and relatively inexpensive monitoring throughout the disease process [10]. These biomarkers include immunoregulatory cells, soluble mediators of inflammation, and a panel of features, such as absolute counts of neutrophils, lymphocytes, platelets, or their combined ratios [11]. Investigating the status and activity of systemic immunity and inflammation is promising because it could provide dynamic profiling of the tumor microenvironment and its effects on immune modulation [12]. Despite these premises, no soluble biomarkers received validation or approval for the management of patients undergoing immune checkpoint blockade.

A novel type of blood-based biomarkers are systemic indicators that are not directly linked to the immune system but can still reflect its activity and the modulatory effects of ICIs [13]. In this context, thyroid hormone balance, defined by the free triiodothyronine (fT3) to free thyroxin (fT4) conversion ratio, has emerged as a strong prognostic factor in patients with advanced colorectal cancer or renal cell carcinoma receiving tyrosine kinase inhibitors [14,15,16]. More recently, the fT3/fT4 ratio showed a significant correlation with survival outcomes in patients treated with immunotherapy for advanced urothelial carcinoma, regardless of other established prognostic factors [17]. The fT3/fT4 ratio is a viable indicator of different deiodinases that modulate the bioavailability of the active form of thyroid hormones at the peripheral tissue level [18]. Growing evidence suggests a direct interplay between this enzyme activity and the immune system [19]. Several components of the innate and adaptive immunity can interfere with the balance of thyroid hormones by regulating deiodinase activity [20]. Considering the well-known effects of ICIs on thyroid function and immune response, it is conceivable that the fT3/fT4 ratio may also change during such treatment [21]. Therefore, beyond its prognostic value, dynamic changes in the conversion ratio could serve as predictive biomarkers during immune checkpoint blockade. Moreover, to the best of our knowledge, the role of the fT3/fT4 ratio in patients receiving ICIs for advanced lung cancer has never been established. Based on these insights, we sought to evaluate the correlation between changes in the fT3/fT4 ratio and survival outcomes of advanced NCLC patients treated with upfront pembrolizumab-based therapy.

## 2. Materials and Methods

### 2.1. Study Design and Patients

The current research relied on a retrospective analysis of patients with advanced NSCLC who were participating in the National Drug Agency’s prospective monitoring program [22]. The study included patients on the basis of consecutive enrollment if they had a histological diagnosis of stage IV NSCLC and an Eastern Cooperative Oncology Group Performance Status (ECOG PS) of 0-2. The treatments consisted of either pembrolizumab administered alone or combined with platinum-based chemotherapy as first-line options. From April 2018 to May 2023, every eligible participant received a minimum of two treatment cycles at our facility. We excluded patients with an unknown PD-L1 tumor proportion score (TPS), sensitizing mutations in the EGFR, BRAF, ALK, or ROS-1 genes, and those who had been exposed to high-dose corticosteroids or other immunosuppressive medications in the 14 days prior to the start of pembrolizumab. Patients with disease recurrence after resection or definitive thoracic radiotherapy were eligible if progression occurred more than 24 weeks after the last perioperative chemotherapy administration. Conversely, previous receipt of anti-PD-(L)1 inhibitors at any time represented an exclusion criterion for the present analysis. We also included patients with metastatic brain involvement in cases where they were asymptomatic or neurologically stable after radiotherapy. In addition, abnormal thyroid-stimulating hormone (TSH) levels and/or levothyroxine replacement therapy at baseline were key exclusion criteria for the purpose of the current research. This research complied with the STROBE guidelines for observational studies [23] and received approval by the relevant Ethics Committee (registration code: Oss-R-281; protocol number: 855/CE Lazio1). We obtained written consent from all patients, which included permission to process and use deidentified personal data for clinical research purposes.

### 2.2. Data Collection and Assessment of Outcomes

The National Drug Agency provided information on demographic, clinical, pathological, and molecular characteristics, as well as data on treatment outcomes in terms of disease response and survival rates [22]. The immunohistochemical evaluation of the PD-L1 TPS was performed in at least 100 viable tumor cells using the 22C3 pharmDx anti-PD-L1 antibody (Agilent Technologies, Inc., Santa Clara, CA, USA) [24]. The institutional database provided peripheral blood test results, including TSH, fT3, and fT4. We referred to the harmonized norms for thyroid function indices (TSH: 0.25–4.94 uIU/mL, fT3: 1.71–3.71 pg/dL, and fT4: 7.00–14.80 pg/dL). The fT3 to fT4 ratio was calculated for each patient before starting pembrolizumab (time point 1) and again after 12 weeks (time point 2). Based on their presumed effect on immune checkpoint blockade efficacy, we also addressed the exposure to the following concomitant drugs: corticosteroids (dose ≥ 10 mg per day of a prednisone equivalent for at least 5-day dosing within the 30 days prior to starting first-line treatment, excluding medications prior to chemotherapy), systemic antibiotics (within 30 days before starting first-line treatment), and acetaminophen (APAP) and proton pump inhibitor (PPI) intake at first-line treatment initiation. The primary purpose of the current analysis was to assess the impact of dynamic changes in the fT3/fT4 ratio on progression-free survival (PFS) and overall survival (OS). According to National Drug Agency guidelines, a baseline disease assessment was performed within four weeks of treatment initiation. These rules also provide for an initial reassessment of the disease between 12 and 16 weeks after the start of treatment and every four to six months thereafter [22]. A blinded radiologist reviewed patients’ radiology records using the Response Evaluation Criteria in Solid Tumors for immunotherapy (iRECIST) [25]. PFS was calculated from the initial administration of pembrolizumab to the date of disease progression. OS was calculated from the initial administration of pembrolizumab until death, regardless of cause. Patients whose disease was not progressive and who were still alive by the last follow-up were censored (as of 31 May 2024).

### 2.3. Statistical Analysis

We analyzed demographic, clinical, and pathological data using descriptive statistics. Statistical distribution indices included a mean with standard deviation (SD) in cases of normality and a median with a 95% confidence interval (CI) or interquartile range (IQR) in cases of skewness. We stratified patients into two subgroups based on baseline fT3 and fT4 values [euthyroid cohort (ETC) defined by fT3 and fT4 concentrations both within the normal range vs. euthyroid sick syndrome cohort (ESC) defined by low fT3 and/or fT4 levels]. We applied propensity score matching (PSM) to adjust for the potential imbalance of baseline covariates between study cohorts. Propensity scores were estimated through a multivariate logistic regression model that accounted for all relevant covariates, including age, sex, ECOG PS, histological subtype, disease extent, specific metastatic involvement (bone, brain, and/or liver), PD-L1 TPS, body mass index (BMI), smoking habits, previous thoracic radiotherapy, lung immune prognostic index (LIPI) score, treatment regimen, and concomitant medications (corticosteroids, APAP, antibiotics, and/or PPI). To ensure sufficient numbers of participants and equal representation in both groups, we employed a one-to-one matching approach using the nearest-neighbor algorithm with a caliper width of 0.2. Comparative assessments were performed before and after PSM to demonstrate successful balancing of baseline risk using Pearson’s χ^2^, Mann–Whitney’s U test, or Kruskal–Wallis test, as appropriate. Pairwise comparisons were carried out using the Wilcoxon signed-rank test. Receiving operating characteristic (ROC) curves were calculated at both time points to assess the reliability of fT3/fT4 distributions in predicting the probability of positive survival outcomes. PFS and OS were estimated using the Kaplan–Meier method and compared applying the log-rank test and Cox’s proportional hazards method. We performed a univariate analysis to determine the association between survival outcomes and fT3/fT4 categories, in addition to the variables used to estimate propensity scores. Covariates that showed any association with a *p* value less than 0.05 were included in the multivariate analysis. Hazard ratios (HRs) with a 95% CI comparing the incidences of disease progression and death were estimated by fitting Cox proportional hazards regression models. All tests performed were two-sided, and the level of statistical significance was set at a *p* value lower than 0.05. SPSS version 23.0 (IBM SPSS Statistics for Windows, Armonk, NY, USA) and GraphPad Prism version 9.0 (GraphPad Software Inc., San Diego, CA, USA) enabled statistical analysis and graphing, respectively. PSM required the application of R software version 4.1.2 and the MatchIt library [26].

## 3. Results

### 3.1. Patient Characteristics

We enrolled 258 consecutive patients who met the inclusion criteria for this analysis. All participants received pembrolizumab as their first-line treatment after the date of official registration for use in clinical practice. Between April 2018 and May 2023, 156 (60.5%) patients with a PD-L1 TPS ≥ 50% were given pembrolizumab as exclusive therapy. Additionally, from November 2019 to May 2023, 102 (39.5%) patients with a PD-L1 TPS < 50% were treated with pembrolizumab in combination with platinum-based chemotherapy. In both treatment settings, all participants had metastatic disease extent and an ECOG PS ranging from 0 to 2. The definition of baseline thyroid function allowed us to identify 88 (34.1%) patients who accounted for the ESC in comparison to all others (ETC). While the clinical and pathological characteristics were evenly distributed between these cohorts, we observed a significant imbalance in terms of pharmacological variables. Specifically, patients in the ETC had a higher prevalence of APAP and PPI intake. Adjustment by PSM resulted in a closely balanced distribution of baseline covariates between the subgroups. After implementing PSM, we finally analyzed a combined group of 88 patients from each cohort. Table 1 details the baseline characteristics of both populations.

### 3.2. Dynamic Changes in Thyroid Function

**Figure 1:**
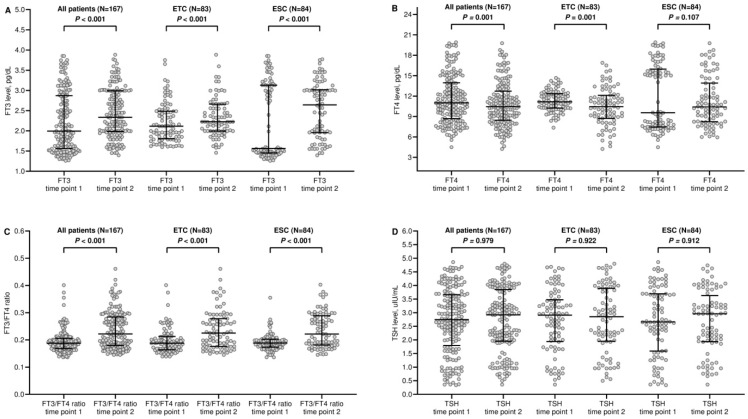
Comparison of scatter plot distributions of thyroid function indices. (**A**) FT3, free triiodothyronine; (**B**) FT4, free thyroxin; (**C**) FT3/FT4, FT3 to FT4 conversion ratio; and (**D**) TSH, thyroid-stimulating hormone. ETC, euthyroid cohort; ESC, euthyroid sick syndrome cohort. Bars represent median values with interquartile range. Time point 1 indicates the assessment before starting pembrolizumab-based therapy. Time point 2 indicates the assessment 12 weeks after the start of pembrolizumab-based therapy.

Before the second time point, nine patients (5.1%) developed clinically relevant hypothyroidism that required levothyroxine supplementation and were excluded from subsequent comparative assessments. Therapy with pembrolizumab resulted in a significant increase in fT3 levels in the whole population of interest and in both cohorts (Figure 1A and Table 2). Conversely, fT4 concentrations decreased significantly in the general group and the ETC (Figure 1B and Table 2). These changes in thyroid hormone balance led to a widespread and statistically significant rise in the fT3/fT4 ratio (Figure 1C and Table 2). It is noteworthy that TSH levels did not vary across all comparisons (Figure 1D and Table 2).

**Figure 2:**
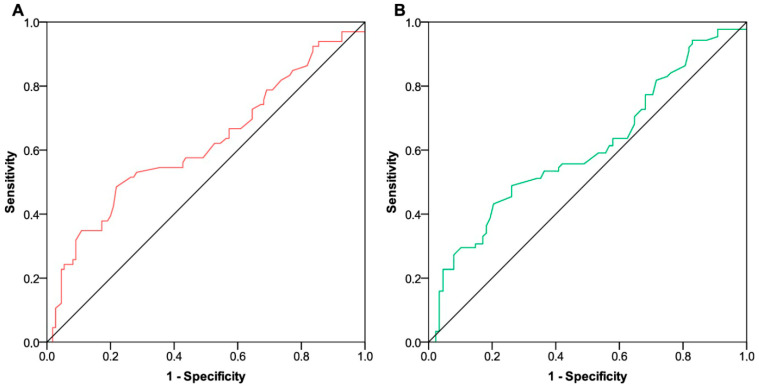
ROC curve analysis of FT3/FT4 ratio on survival outcomes (time point 1). (**A**) ROC curve analysis showing the performance of fT3/fT4 ratio distributions in predicting 1-year progression-free survival at time point 1 [AUC 0.61 (95% CI 0.52–0.70; *p* = 0.009)]. (**B**) ROC curve analysis showing the performance of fT3/fT4 ratio distributions in predicting 1-year overall survival at time point 1 [AUC 0.60 (95% CI 0.52–0.68; *p* = 0.017)]. ROC, receiving operating characteristic; FT3, free triiodothyronine; FT4, free thyroxin; AUC, area under the curve; CI, confidence interval. Time point 1 indicates the assessment before starting pembrolizumab-based therapy.

**Figure 3:**
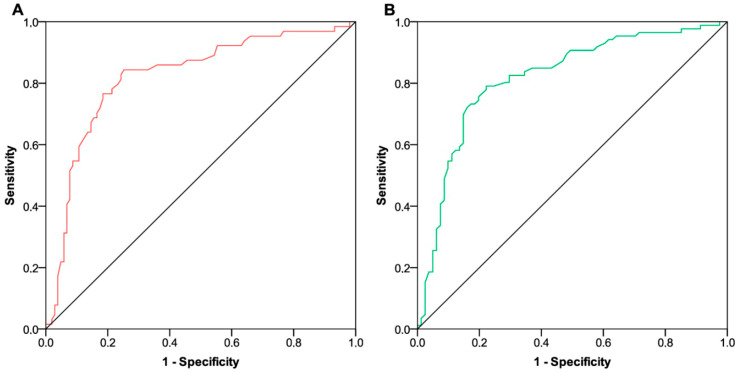
ROC curve analysis of FT3/FT4 ratio on survival outcomes (time point 2). (**A**) ROC curve analysis showing the performance of fT3/fT4 ratio distributions in predicting 1-year progression-free survival at time point 2 [AUC 0.82 (95% CI 0.75–0.89; *p* < 0.001)]. (**B**) ROC curve analysis showing the performance of fT3/fT4 ratio distributions in predicting 1-year overall survival at time point 2 [AUC 0.81 (95% CI 0.75–0.88; *p* < 0.001)]. ROC, receiving operating characteristic; FT3, free triiodothyronine; FT4, free thyroxin; AUC, area under the curve; CI, confidence interval. Time point 2 indicates the assessment 12 weeks after the start of pembrolizumab-based therapy.

All patients relevant to the efficacy analysis received at least two cycles of treatment and were therefore evaluable for objective response at first disease restaging. We described an overall response rate (ORR) of 35.3% (95% CI 28.1–43.1) [resulting from four complete (2.4%) and fifty-five partial responses (32.9%)], 49 disease stabilizations (28.1%, 95% CI 21.5–35.6), and 61 disease progressions (36.5%, 95% CI 29.2–44.3). Among the progressing patients, six (3.6%) had already experienced disease progression after the second or third course of treatment and thus before the expected time point for restaging. The baseline fT3/fT4 ratio did not differ between patients who achieved an ORR [median value 0.19 (IQR 0.17–0.22)] compared with all others [median value 0.18 (IQR 0.16–0.19); *p* = 0.930]. Conversely, the post-treatment fT3/fT4 ratio was significantly higher in patients showing an ORR [median value 0.27 (IQR 0.22–0.30)] than in the remaining subgroup [median value 0.19 (IQR 0.17–0.25); *p* < 0.001]. ROC curve analyses relied on 1-year PFS and OS rates as state variables indicative of a positive outcome. The area under the curve (AUC) values related to fT3/fT4 ratio distributions were not reliable at the first time point (Figure 2A,B). Assessments at the second time point revealed a significant and clinically relevant association between fT3 to fT4 conversion ratios and both PFS and OS outcomes (Figure 3A,B). The application of Youden’s index identified an optimal threshold value of 0.227 (with a sensitivity of 0.84 and specificity of 0.74) for PFS and 0.220 (with a sensitivity of 0.79 and specificity of 0.77) for OS. In both instances, we stratified patients into two subgroups characterized by low and high fT3/fT4 ratios.

### 3.3. Survival Outcomes

**Figure 4:**
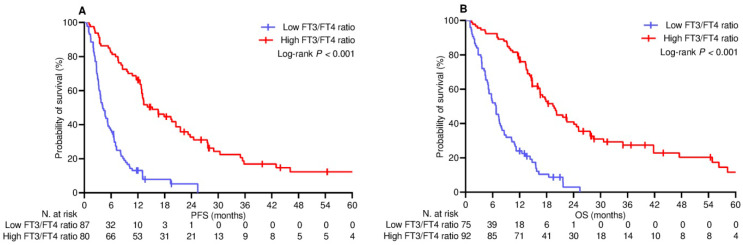
Survival analysis. (**A**) PFS, progression-free survival; (**B**) OS, overall survival; FT3, free triiodothyronine; FT4, free thyroxine.

The median follow-up duration was 20.2 (95% CI 16.1–24.6) months in the PSM-adjusted population relevant to survival analysis. At the cut-off date, 24 (14.4%) patients did not experience any disease progression, while 33 (19.8%) patients were censored without any events relevant to survival. The median PFS and OS were 7.6 (95% CI 6.0–9.3) months and 13.4 (95% CI 11.1–15.7) months, respectively, in the whole population. The median PFS in the low and high fT3/fT4 ratio subgroups was 4.1 (95% CI 3.0–5.1) months and 15.3 (95% CI 10.3–20.1) months, respectively [*p* < 0.001; HR 4.25 (95% CI 2.86–6.17); Figure 4A]. The median OS in the low and high fT3/fT4 ratio subgroups was 6.7 (95% CI 4.9–8.5) months and 19.6 (95% CI 16.4–22.8) months, respectively [*p* < 0.001; HR 4.36 (95% CI 2.98–6.39); Figure 4B]. On univariate analysis, metastatic bone involvement, BMI less than 25 kg/m^2^, nonsmoking habits, LIPI score greater than 0, and exposure to corticosteroids or antibiotics were the other covariates associated with worse PFS. With the exception of bone metastasis and smoking habits, the same factors also correlated with poorer OS (Table 3). The multivariable Cox hazard regression model confirmed that corticosteroid intake, LIPI classification, and fT3/fT4 ratio had an independent effect on both PFS and OS (Table 4).

## 4. Discussion

The current research looked at whether dynamic changes in the fT4 to fT4 conversion ratio during pembrolizumab-based treatment would affect the survival of patients with advanced NSCLC. We found that the distribution of the fT3/fT4 ratio preceding the onset of immune checkpoint blockade did not have an impact on PFS or OS. However, our multivariate analysis revealed that patients characterized by a higher ratio after 12 weeks of treatment experienced a significant reduction in the risk of disease progression and death. These findings, which we believe to be unprecedented, raise two major points for discussion.

The study relies on a research methodology that involves real-life data arising from the current practice of a single center. Real-world studies have been proven to be valuable in cancer research because they can address issues that would be difficult to examine through prospective investigations [27]. In this regard, we performed a retrospective analysis to address the unanswered question of whether changes in thyroid hormone balance could affect outcomes during immune checkpoint blockade. First, we sought to achieve optimal prognostic balance for the entire dataset through appropriate PSM. Following best practice guidelines for medical research, this weighting was based on all relevant clinical, pathological, and pharmacological covariates [28]. A second aspect supporting the reliability of our data is their close adherence to the National Drug Agency registry. This institution ensures the reimbursement of high-cost drugs, including the pembrolizumab under study, through prospective monitoring [22].

The first controversial issue concerns the lack of the prognostic potential of the baseline fT3/fT4 ratio in advanced NSCLC. The mutual balance between the active forms of thyroid hormones has emerged as a reliable marker of peripheral deiodinase activity. Three different isoforms of this family of enzymes are involved in the biotransformation of the inactive T4 precursor produced by the thyroid gland into active T3 (deiodinases 1 [D1] and 2 [D2]) or into inactive reverse T3 from T4 and T2 from T3 (deiodinase 3 [D3]) [29]. Several pathological conditions characterized by chronic metabolic impairment can result in decreased D1 and D2 activity and increased D3 activity. The consequent downregulation of fT3 bioavailability in the absence of hypothalamic or pituitary dysfunction (normal TSH) defines “euthyroid sick syndrome” (ESS) or “nonthyroidal illness syndrome” [18]. Clinical evidence has identified ESS as negative prognostic factors in a variety of advanced chronic diseases [30,31,32]. These studies have also suggested fT3 imbalance as an indirect marker of global frailty, defined by the co-presence of disturbances in nutritional status, multiple organ impairment, and chronic inflammation [33,34]. Based on these insights, subsequent clinical research has confirmed that a low fT3/fT4 ratio at baseline is a strong prognostic factor in several types of cancer patients on active treatment [14,15,16,17]. The findings of the present research are not consistent with the latter evidence, and several reasons may account for this discrepancy. Previous studies relied on the implication that a lower fT3/fT4 ratio at baseline underlies metabolic alterations which may adversely affect prognosis [35,36]. The clinical context of patients with untreated advanced NSCLC who are eligible to receive upfront immunotherapy and platinum-based chemotherapy may be profoundly different. In this condition, the presence of organic disorders, such as cachexia, sarcopenia, renal or hepatic impairment, and chronic inflammatory response, appears less likely and relevant than in heavily pretreated colorectal [14,15] or urothelial cancer patients [17]. Only one study has so far evaluated the prognostic significance of ESS in lung cancer [37]. Although ESS was significantly identified as an unfavorable prognostic factor, the authors included a mixed case series of patients with non-small cell and small cell histology and early, locally advanced, and metastatic stages, making any comparison inconclusive. In addition, all studies involving actively treated cancer patients stratified participants according to baseline values of the fT3/fT4 ratio without balancing established prognostic factors. Similarly, the categorization of patients relied on an arbitrary distribution in tertiles of fT3/fT4 values without a prior assessment of appropriate cutoff points. We performed a stratification of patients based on thyroid functional class, weighing all potential prognostic factors through close PSM. We also evaluated the threshold values of fT3/fT4 distribution according to predefined survival outcomes. Unlike previous studies, this analysis methodology may have limited the impact of selection bias and reduced the prognostic potential of the baseline fT3/fT4 ratio.

The second issue concerns the dynamic changes in the fT3/fT4 ratio following immune checkpoint blockade. We observed a significant variation in the conversion ratio, supported by the increase and decrease in fT3 and fT4 concentrations, respectively. TSH levels, on the other hand, showed no significant changes. The latter finding is consistent with available evidence suggesting that TSH values usually remain within the normal range during chronic disease for several reasons (pituitary dysfunction, a decreased production of thyrotropin-releasing hormone by the hypothalamus, and reduced TSH pulsatility) [18,38]. Since patients with a higher fT3/fT4 ratio achieve a significant and independent reduction in the risk of disease progression and mortality, incremental change seems to gain predictive potential for treatment efficacy. These results are unprecedented and their interpretation is evidently challenging. Because patients who showed immune-related thyroid toxicity within the second time point were excluded from the survival analysis owing to direct effects on thyroid hormone levels and survival outcomes [39], we argued that changes in the reciprocal balance between fT3 and fT4 may depend on the modulation of deiodinase activity. This favorable variation during treatment, resulting in the recovery or attenuation of the effects of ESS, could underlie a positive anti-tumor immune response induced by ICIs. The interaction between thyroid hormones and the immune system involves a bidirectional crosstalk [40]. While several pathological conditions or autoimmunity interfere with thyroid hormone homeostasis, their mutual balance has been described as regulating innate and adaptive immune cells [41]. Our multivariate analysis identified the level of fT3/fT4 ratio during treatment and baseline LIPI classification as the strongest predictors of survival. The LIPI score may indicate a pro-inflammatory state in the tumor microenvironment, representing the balance between neutrophils and lymphocytes as well as the extent of tumor metabolic activity through LDH levels [42]. Baseline categorization according to the LIPI score showed significant predictive potential in patients with advanced disease receiving ICIs or chemotherapy, regardless of cancer type [43]. Based on these suggestions, we can suppose an interaction between fT3/fT4 ratio dynamics and LIPI scoring that can reflect the overall immune response of patients with advanced NSCLC on the immune checkpoint blockade. Although this hypothesis is consistent with similar assumptions [44], the lack of clinical evidence does not allow further insights to be drawn.

Our study has several limitations that need to be recognized. Firstly, the research design was based on a retrospective analysis conducted at a single center. Although we used a prospective government registry as data source and had predefined evaluation schedules and uniform criteria for treatment decisions, selection bias could not be excluded. We did try to minimize this by enrolling consecutive patients and using comprehensive criteria for PSM, but confounding may have had a not negligible relevance. Secondly, our assessments of disease response and treatment failure relied on radiological examinations that were blinded but not independent. This implies the possibility that the efficacy of immune checkpoint blockade in terms of PFS may have been overestimated. Thirdly, we referred to internal records to gather retrospective data on laboratory test results. While this method is convenient, it may not be as accurate as data obtained directly from a clinical trial. Moreover, as much as we tried to limit the effects of immune-related adverse events on the change in the fT3/fT4 ratio by excluding from survival analysis patients developing clinically relevant hypothyroidism, we cannot rule out the impact of subclinical toxicities. Lastly, we cannot overlook alpha-risk inflation that may arise from multiple comparisons. While we needed to use multivariate regressions to analyze our novel experimental data, this increased the chances of obtaining false-positive results.

## 5. Conclusions

In advanced NSCLC undergoing upfront pembrolizumab-based therapy, we identified changes in the fT3/fT4 ratio during treatment as an independent predictive biomarker. Since the combination of multiple biomarkers may be necessary for a more accurate prediction of the efficacy of the immune checkpoint blockade, a longitudinal assessment of dynamic changes in the thyroid hormone balance could make a contribution in this therapeutic setting. However, it is essential to acknowledge the intrinsic constraints of this study and the lack of sufficient evidence for a comprehensive comparison. Therefore, caution should be exercised, and further validation in independent series is mandatory.

## Figures and Tables

**Table 1:** Patient characteristics.

Variable	Unadjusted Population				PSM-Adjusted Population			
	All Patients (*n* = 258)	ETC (*n* = 170)	ESC (*n* = 88)	*p* Value	All Patients (*n* = 176)	ETC (*n* = 88)	ESC (*n* = 88)	*p* Value
Age								
- Mean (SD), years	70.0 (8.4)	70.0 (7.9)	69.5 (9.0)	0.332	68.4 (8.7)	68.3 (8.7)	68.6 (8.8)	0.689
- ≥70 years	136 (52.7%)	91 (53.5%)	45 (51.1%)	0.715	88 (50.0%)	43 (48.9%)	45 (51.1%)	0.763
Sex				0.69				0.744
- Female	78 (30.2%)	50 (29.4%)	28 (31.8%)		54 (30.7%)	26 (29.5%)	28 (31.8%)	
- Male	180 (69.8%)	120 (70.6%)	60 (68.2%)		122 (69.3%)	62 (70.5%)	60 (68.2%)	
ECOG PS				0.986				0.579
- 0 or 1	208 (80.6%)	137 (80.6%)	71 (80.7%)		139 (79.0%)	68 (77.3%)	71 (80.7%)	
- 2	50 (19.4%)	33 (19.4%)	17 (19.3%)		37 (21.0%)	20 (22.7%)	17 (19.3%)	
Histologic subtype				0.545				0.604
- Nonsquamous	202 (78.3%)	135 (79.4%)	67 (76.1%)		131 (74.4%)	64 (72.7%)	67 (76.1%)	
- Squamous	56 (21.7%)	35 (20.6%)	21 (23.9%)		45 (25.6%)	24 (27.3%)	21 (23.9%)	
No. of metastatic sites				0.611				0.444
- ≤2	138 (53.5%)	89 (52.4%)	49 (55.7%)		103 (58.5%)	54 (61.4%)	49 (47.6%)	
- >2	120 (46.5%)	81 (47.6%)	39 (44.3%)		73 (41.5%)	34 (38.6%)	39 (44.3%)	
Bone metastasis	53 (20.5%)	35 (20.6%)	18 (20.5%)	0.98	31 (17.6%)	13 (14.8%)	18 (20.5%)	0.322
Brain metastasis	58 (22.5%)	37 (21.8%)	21 (23.9%)	0.702	43 (24.4%)	22 (25.0%)	21 (23.9%)	0.861
Liver metastasis	28 (10.9%)	19 (11.2%)	9 (10.2%)	0.816	16 (9.1%)	7 (8.0%)	9 (10.2%)	0.6
PD-L1 TPS				0.116				0.127
- <1%	61 (23.6%)	46 (27.0%)	15 (17.0%)		48 (27.3%)	30 (34.1%)	18 (20.4%)	
- ≥1% and ≤49%	41 (15.9%)	23 (13.5%)	18 (20.4%)		19 (10.8%)	7 (7.9%)	12 (13.6%)	
- ≥50%	156 (60.4%)	101 (59.4%)	55 (62.5%)		109 (61.9%)	54 (61.4%)	55 (62.5%)	
BMI								
- Mean (SD), kg/m^2^	25.1 (4.80)	24.8 (5.0)	25.6 (4.5)	0.635	26.0 (4.8)	26.2 (5.2)	25.8 (4.4)	0.776
- ≥25 kg/m^2^	130 (50.4%)	83 (48.8%)	47 (53.4%)	0.485	92 (52.3%)	45 (51.1%)	47 (53.4%)	0.763
Smoking habits				0.943				0.799
- Never	23 (8.9%)	15 (8.8%)	8 (9.1%)		17 (9.7%)	9 (10.2%)	8 (9.1%)	
- Ever	235 (91.1%)	155 (91.2%)	80 (90.9%)		159 (90.3%)	79 (89.8%)	80 (90.9%)	
Previous thoracic RT	35 (13.6%)	22 (12.9%)	13 (14.8%)	0.684	26 (14.8%)	13 (14.8%)	13 (14.8%)	1
LIPI score				0.999				0.953
- 0	100 (38.8%)	66 (38.8%)	34 (38.6%)		70 (39.8%)	36 (40.9%)	34 (38.6%)	
- 1	88 (34.1%)	58 (34.1%)	30 (34.1%)		59 (33.5%)	29 (33.0%)	30 (34.1%)	
- 2	70 (27.1%)	46 (27.1%)	24 (27.3%)		47 (26.7%)	23 (26.1%)	24 (27.3%)	
First-line therapy				0.887				0.958
- Only pembrolizumab	156 (60.5%)	101 (59.4%)	55 (62.5%)		109 (61.9%)	54 (61.4%)	55 (62.5%)	
- Pemetrexed-based	84 (32.6%)	57 (33.5%)	27 (30.7%)		54 (30.7%)	27 (30.7%)	27 (30.7%)	
- Paclitaxel-based	18 (7.0%)	12 (7.1%)	6 (6.8%)		13 (7.4%)	7 (8.0%)	6 (6.7%)	
Corticosteroids ^a^	103 (39.9%)	66 (38.8%)	37 (42.0%)	0.616	72 (40.9%)	35 (39.8%)	37 (42.0%)	0.759
APAP ^b^	101 (39.1%)	74 (43.5%)	27 (30.6%)	0.045	53 (28.7%)	26 (29.5%)	27 (30.7%)	0.869
Systemic antibiotics ^c^	58 (22.5%)	40 (23.5%)	18 (20.5%)	0.575	33 (18.8%)	15 (17.0%)	18 (20.5%)	0.562
PPI ^d^	98 (38.0%)	72 (42.3%)	26 (29.5%)	0.044	46 (26.1%)	20 (22.7%)	26 (29.5%)	0.303
FT3, pg/dL				0.091				0.096
- Median (IQR)	2.05 (1.57–2.93)	2.16 (1.85–2.61)	1.59 (1.47–3.05)		1.99 (1.56–2.87)	2.11 (1.80–2.48)	1.56 (1.45–3.12)	
FT4, pg/dL				0.451				0.519
- Median (IQR)	10.97 (8.53–13.75)	11.04 (10.27–12.48)	9.46 (7.27–16.11)		11.01 (8.67–13.94)	11.15 (10.33–12.33)	9.55 (7.46–16.23)	
TSH, uIU/mL								
- Median (IQR)	2.76 (1.74–3.72)	2.82 (1.87–3.61)	2.61 (1.56–3.72)	0.897	2.74 (1.79–3.66)	2.90 (1.93–3.47)	2.66 (1.58–3.69)	0.866

PSM, propensity score matching; ETC, euthyroid cohort; ESC, euthyroid sick syndrome cohort; SD, standard deviation; ECOG PS, Eastern Cooperative Oncology Group Performance Status; PD-L1 TPS, programmed cell death ligand-1 tumor proportion score; BMI, body mass index; RT, radiotherapy; LIPI, lung immune prognostic index; APAP, acetaminophen; PPI, proton pump inhibitors; FT3, free triiodothyronine; FT4, free thyroxin; IQR, interquartile range; TSH, thyroid-stimulating hormone. ^a^ Corticosteroids indicate intake of prednisone equivalent ≥ 10 mg daily for at least 5 days within the 30 days prior to the start of treatment (excluding premedication for chemotherapy); ^b^ APAP indicates a therapeutic intake of at least 1000 mg per day for more than 24 h during the 30 days prior to the start of treatment; ^c^ systemic antibiotics indicate a therapeutic intake in the 30 days prior to the start of treatment; ^d^ PPI indicates any intake at the start of treatment.

**Table 2:** Comparison of changes in thyroid function.

	Time Point 1	Time Point 2	*p* Value
PSM-adjusted population (*n* = 167)			
FT3, pg/dL - median (IQR)	2.05 (1.55–2.87)	2.33 (1.98–2.99)	<0.001
FT4, pg/dL - median (IQR)	11.12 (8.64–13.94)	10.44 (8.45–12.72)	0.001
TSH, uIU/mL - median (IQR)	2.75 (1.72–3.66)	2.91 (1.94–3.84)	0.979
FT3/FT4 ratio	0.18 (0.16–0.20)	0.22 (0.17–0.28)	<0.001
ETC (*n* = 83)			
FT3, pg/dL - median (IQR)	2.11 (1.85–2.51)	2.22 (1.99–2.66)	<0.001
FT4, pg/dL - median (IQR)	11.25 (10.24–12.42)	10.44 (8.72–12.11)	0.001
TSH, uIU/mL - median (IQR)	2.94 (1.74–3.58)	2.85 (1.94–3.89)	0.922
FT3/FT4 ratio	0.18 (0.16–0.21)	0.22 (0.17–0.27)	<0.001
ESC (*n* = 84)			
FT3, pg/dL - median (IQR)	1.56 (1.45–3.15)	2.64 (1.95–3.01)	<0.001
FT4, pg/dL - median (IQR)	9.55 (7.45–16.23)	10.41 (8.23–13.90)	0.107
TSH, uIU/mL - median (IQR)	2.66 (1.54–3.69)	2.95 (1.93–3.62)	0.912
FT3/FT4 ratio	0.18 (0.17–0.20)	0.22 (0.18–0.28)	<0.001

PSM, propensity score matching; FT3, free triiodothyronine; FT4, free thyroxin; IQR, interquartile range; TSH, thyroid-stimulating hormone; ETC, euthyroid cohort; ESC, euthyroid sick syndrome cohort. Time point 1 indicates the assessment before starting pembrolizumab-based therapy; time point 2 indicates the assessment 12 weeks after the start of pembrolizumab-based therapy.

**Table 3:** Univariate analysis of survival.

Covariate	Progression-Free Survival		Overall Survival	
	HR (95% CI)	*p* Value	HR (95% CI)	*p* Value
Age				
- <70 years	1	-	1	-
- ≥70 years	0.82 (0.59–1.14)	0.246	0.81 (0.58–1.14)	0.241
Sex				
- Female	1	-	1	-
- Male	0.91 (0.64–1.31)	0.628	0.94 (0.65–1.35)	0.742
ECOG PS				
- 0–1	1	-	1	-
- 2	1.48 (0.98–2.22)	0.057	1.46 (0.96–2.23)	0.074
Histologic subtype				
- Nonsquamous	1	-	1	-
- Squamous	0.82 (0.56–1.20)	0.321	0.93 (0.63–1.38)	0.747
No. of metastatic sites				
- ≤2	1	-	1	-
- >2	1.22 (0.87–1.71)	0.233	1.23 (0.87–1.75)	0.229
Bone metastasis				
- No	1	-	1	-
- Yes	1.74 (1.15–2.65)	0.009	1.46 (0.93–2.28)	0.093
Brain metastasis				
- No	1	-	1	-
- Yes	0.89 (0.60–1.32)	0.583	0.88 (0.58–1.33)	0.559
Liver metastasis				
- No	1	-	1	-
- Yes	1.44 (0.84–2.47)	0.179	1.27 (0.72–2.27)	0.402
PD-L1 TPS				
- ≥50%	1	-	1	-
- <50%	1.06 (0.75–1.50)	0.726	0.94 (0.65–1.35)	0.758
BMI				
- ≥25 kg/m^2^	1	-	1	-
- <25 kg/m^2^	1.47 (1.05–2.05)	0.022	1.49 (1.06–2.10)	0.022
Smoking habits				
- Ever	1	-	1	-
- Never	1.95 (1.10–3.48)	0.022	1.39 (0.78–2.47)	0.257
Previous thoracic RT				
- No	1	-	1	-
- Yes	0.81 (0.50–1.30)	0.389	0.74 (0.45–1.21)	0.239
LIPI score				
- 0	1	-	1	-
- 1	4.63 (2.90–7.40)	<0.001	7.67 (4.43–13.25)	<0.001
- 2	14.96 (8.87–25.29)	<0.001	32.00 (17.01–60.22)	<0.001
First-line therapy				
- Only pembrolizumab	1	-	1	-
- Pemetrexed-based	1.07 (0.74–1.55)	0.703	0.91 (0.61–1.34)	0.642
- Paclitaxel-based	1.02 (0.52–1.97)	0.954	1.09 (0.56–2.13)	0.782
Corticosteroids ^a^				
- No	1	-	1	-
- Yes	2.22 (1.58–3.12)	<0.001	2.15 (1.52–3.05)	<0.001
APAP ^b^				
- No	1	-	1	-
- Yes	1.29 (0.91–1.83)	0.149	1.20 (0.83–1.73)	0.312
Systemic antibiotics ^c^				
- No	1	-	1	-
- Yes	2.04 (1.35–3.07)	0.001	2.08 (1.37–3.16)	0.001
PPI ^d^				
- No	1	-	1	-
- Yes	0.91 (0.62–1.32)	0.626	0.91 (0.61–1.34)	0.644
On treatment FT3/FT4 ratio				
- High	1	-	1	-
- Low	4.25 (2.86–6.17)	<0.001	4.36 (2.98–6.39)	<0.001

PFS, progression-free survival; CI, confidence interval; HR, hazard ratio; ECOG PS, Eastern Cooperative Oncology Group Performance Status; PD-L1 TPS, programmed cell death ligand-1 tumor proportion score; BMI, body mass index; RT, radiotherapy; LIPI, lung immune prognostic index; APAP, acetaminophen; PPI, proton pump inhibitors; FT3, free triiodothyronine; FT4, free thyroxin. ^a^ Corticosteroids indicate intake of prednisone equivalent ≥ 10 mg daily for at least 5 days within the 30 days prior to the start of treatment (excluding premedication for chemotherapy); ^b^ APAP indicates a therapeutic intake of at least 1000 mg per day for more than 24 h during the 30 days prior to the start of treatment; ^c^ systemic antibiotics indicate a therapeutic intake in the 30 days prior to the start of treatment; ^d^ PPI indicates any intake at the start of treatment.

**Table 4:** Multivariate analysis of survival.

Covariate	Progression-Free Survival		Overall Survival	
	HR (95% CI)	*p* Value	HR (95% CI)	*p* Value
Bone metastasis			-	-
- No	1	-		
- Yes	0.99 (0.64–1.52)	0.972		
BMI				
- ≥25 kg/m^2^	1	-	1	-
- <25 kg/m^2^	1.29 (0.90–1.84)	0.156	1.41 (0.98–2.02)	0.06
Smoking habits			-	-
- Ever	1	-		
- Never	2.22 (1.21–4.05)	0.009		
LIPI score				
- 0	1	-	1	-
- 1	3.50 (2.15–5.71)	<0.001	5.72 (3.29–9.93)	<0.001
- 2	11.50 (6.52–20.31)	<0.001	21.71 (10.94–43.18)	<0.001
Corticosteroids ^a^				
- No	1	-	1	-
- Yes	1.72 (1.18–2.51)	0.005	1.70 (1.17–2.47)	0.005
Systemic antibiotics ^b^				
- No	1	-	1	-
- Yes	1.31 (0.84–2.03)	0.23	1.21 (0.77–1.90)	0.401
On treatment FT3/FT4 ratio				
- High	1	-	1	-
- Low	2.51 (1.66–3.78)	<0.001	2.18 (1.43–3.34)	<0.001

HR, hazard ratio; CI, confidence interval; BMI, body mass index; LIPI, lung immune prognostic index; FT3, free triiodothyronine; FT4, free thyroxin. ^a^ Corticosteroids indicate intake of prednisone equivalent ≥ 10 mg daily for at least 5 days within the 30 days prior to the start of treatment (excluding premedication for chemotherapy); ^b^ systemic antibiotics indicate a therapeutic intake in the 30 days prior to the start of treatment.

## Data Availability

The datasets generated and analyzed during the current study are available from the corresponding author on reasonable request.
